# ORF-Interrupting Mutations in Monkeypox Virus Genomes from Washington and Ohio, 2022

**DOI:** 10.3390/v14112393

**Published:** 2022-10-29

**Authors:** Jaydee Sereewit, Nicole A. P. Lieberman, Hong Xie, Shah A. K. Mohamed Bakhash, B. Ethan Nunley, Benjamin Chung, Margaret G. Mills, Pavitra Roychoudhury, Alexander L. Greninger

**Affiliations:** 1 Department of Laboratory Medicine and Pathology, University of Washington Medical Center, Seattle, WA 98195, USA; 2 Vaccine and Infectious Disease Division, Fred Hutchinson Cancer Research Center, Seattle, WA 98109, USA

**Keywords:** monkeypox virus, serology, essential gene, genomic deletion, B21R

## Abstract

Monkeypox virus, the causative agent of the 2022 monkeypox outbreak, is a double-stranded DNA virus in the *Orthopoxvirus* genus of the *Poxviridae* family. Genes in terminal regions of *Orthopoxvirus* genomes mostly code for host-pathogen interaction proteins and are prone to selective pressure and modification events. Using viral whole genome sequencing, we identified twenty-five total clinical samples with ORF-disrupting mutations, including twenty samples encoding nonsense mutations in MPXVgp001/191 (OPG001), MPXVgp004/188 (OPG015), MPXVgp010 (OPG023), MPXVgp030 (OPG042), MPXVgp159 (OPG0178), or MPXVgp161 (OPG181). Additional mutations include a frameshift leading to an alternative C-terminus in MPXVgp010 (OPG023) and an insertion in an adenine homopolymer at the beginning of the annotated ORF for MPXVgp153 (OPG151), encoding a subunit of the RNA polymerase, suggesting the virus may instead use the start codon that encodes Met9 as annotated. Finally, we detected three samples with large (>900 bp) deletions. These included a 913 bp deletion that truncates the C-terminus of MPXVgp010 (OPG023); a 4205 bp deletion that eliminates MPXVgp012 (OPG025), MPXVgp013 (OPG027), and MPXVgp014 (OPG029) and truncates MPXVgp011 (OPG024; D8L) and MPXVgp015 (OPG030); and a 6881 bp deletion that truncates MPXVgp182 (OPG210) and eliminates putative ORFs MPXVgp184, MPXVgp185 (OPG005), and MPXVgp186, as well as MPXVgp187 (OPG016), and MPXVgp188 (OPG015) from the 3’ ITR only. MPXVgp182 encodes the monkeypox-specific, highly immunogenic surface glycoprotein B21R which has been proposed as a serological target. Overall, we find greater than one-tenth of our sequenced MPXV isolates have at least one gene inactivating mutation and these genes together comprised greater than one-tenth of annotated MPXV genes. Our findings highlight non-essential genes in monkeypox virus that may be evolving as a result of selective pressure in humans, as well as the limitations of targeting them for therapeutics and diagnostic testing.

## 1. Introduction

Monkeypox virus (MPXV) is a linear double-stranded DNA virus in the *Orthopoxvirus* genus of the *Poxviridae* family [1]. The MPXV genome is approximately 197 kb in length and contains approximately 190 genes, with inverted terminal repeats (ITR) flanking the terminal ends [2]. Like all *Orthopoxvirus* species, essential viral replication and assembly genes are in the highly conserved central core region (nucleotide positions 56 kbp-120 kbp) [2], while genes in the terminal regions mostly code for proteins that interact with the host immune system and are prone to diversifying selection [3]. Single base changes, small insertions/deletions, gene loss, and horizontal gene transfer are the major mechanisms that contributed to variations in the terminal regions of *Orthopoxvirus* genomes and serve as determinants of host tropism [4]. Orthopoxviruses have generally been thought to have low mutation rates at an estimated 1–2 substitutions per genome per year [5]. However, the 2022 MPXV outbreak strains contain far more mutations than expected, potentially driven by APOBEC3 editing [6,7]. From endemic African strains to the current outbreak strains, deletions ranging from 500 bp to 15 kbp have been reported [2,6,8,9,10], some of which have been associated with virulence and fitness. For example, deletion of ortholog of the vaccinia virus C3L complement-binding protein is thought to decrease virulence of the West African strains relative to Central African strains [2,11,12].

Here, we report twenty-five total clinical MPXV samples from the states of Washington and Ohio with open reading frame (ORF)-disrupting mutations, including one 913 bp deletion in MPXVgp010, one 4205 bp deletion spanning from MPXVgp011 to MPXVgp015, and one 6881 bp deletion adjacent to and partially encompassing the 3’ ITR region. The premature stop codon mutations occur in non-essential genes in the terminal regions, except for the DNA-dependent RNA polymerase subunit rpo132 which likely uses an alternative start codon. The 7 kb deletion eliminates MPXVgp184 to MPXVgp188 and truncates MPXVgp182 (OPG210), which encodes the highly immunogenic surface glycoprotein B21R and was proposed as an antibody target for a serological diagnostic specific for MPXV [13]. 

## 2. Materials and Methods

### 2.1. DNA Extraction, Library Preparation, and Whole Genome Sequencing

Samples were collected as lesion swabs in viral transport medium [14]. DNA was extracted using the Roche MagNA Pure 96 DNA and viral NA Small Volume Kit. 100 µL of the sample was mixed with 100 µL Qiagen AL Buffer and used for extraction with a 100 µL elution. The Ct value for each sample was determined by qPCR of the F3L locus [15] and samples with Ct values < 30 were selected for whole genome sequencing. Sequencing libraries were constructed using the Illumina Nextera Flex DNA Prep M kit following manufacturer’s instructions. Briefly, the extracted DNA was tagmented for 14 min to fragment genomic DNA and add sequencing adapters, followed by 16 to 21 cycles of PCR amplification. The sequencing libraries were cleaned using 0.8× volume of AMPure beads (Beckman Coulter, San Diego, CA, USA) before hybridization capture using a custom IDT xGen NGS Hybridization Capture panel based on the MPXV MA001 genome (ON563414.2) with 1× tiling density. The resulting libraries were sequenced on an Illumina Nextseq2000 instrument using a P1 Reagent Kit (2 × 150 cycles) format. Sequencing libraries from samples containing the mutations described in this paper were prepared and sequenced twice to control library generation artefacts. All genomic positions are defined relative to the reference strain MA001. 

### 2.2. MPXV Bioinformatic Analysis

Paired-end raw reads were adapter- and quality-trimmed with Trimmomatic v0.39 [16]. Unpaired reads and reads shorter than 120 bp were discarded. Trimmed reads were aligned to the 2022 MPXV outbreak reference strain MA001 ON563414.3 using bbmap v38.96 [17], and duplicated reads discarded. Ambiguously mapped reads were randomly assigned to one of the top-scoring sites to give the inverted terminal repeats regions even coverage. The consensus genome was generated by three iterations of consensus calling using Samtools mpileup v1.15 [18] and iVar consensus v1.3.1 [19] with a minimum base quality of phred score 15, a minimum frequency threshold of 0.6, and a minimum depth of 5, followed by remapping reads to the newly generated consensus. After each iteration, any leading or trailing Ns were removed. The full consensus calling pipeline can be found at https://github.com/greninger-lab/revica/releases/tag/v1.2.1, accessed on 5 October 2022. The quality of consensus genome was assessed by manual inspection of the alignment of the trimmed reads to the consensus genome. Additionally, phylogenetic analysis using the Nextstrain v4.2.0 [20] monkeypox workflow (https://github.com/nextstrain/monkeypox, accessed on 5 October 2022) was performed on 25 samples containing ORF-disrupting mutations and 129 other samples sequenced by our laboratory. In samples with a mutation that resulted in an open reading frame disruption due to nonsense or frameshift mutations, we interrogated the Pfam v33.1 database [21] using the NCBI Conserved Domains interface [22]. Signal sequences were predicted by SignalP6.0 [23]. Sequencing reads and consensus assemblies are available from NCBI BioProject PRJNA862948 and consensus assemblies are published on GenBank (Supplementary Table S1).

### 2.3. Confirmatory Deletion PCRs and ddPCR

To confirm deletions, primer sets were designed that flanked the respective deletion for each of samples: WA-UW-074978, MPXVgp182-F 5′-CTCCATTGAGTGTAAAATTCATG-3′, MPXVgp189-R 5′-GATTGTGCGCATCGTTAACG-3′; WA-UW-096851, MPXVgp011-F 5′-ATCCTCCTCCCCACATTCCAC-3′, MPXVgp015-R 5′-CGGACCCGTGTACTGTCTTT-3′; and WA-UW-082786, MPXVgp010-F 5′-GCGTTGACTTATGGACTCTGG-3′, MPXVgp010-R 5′-GGCTCTACTAGAAGCTACTGG-3′. Each 25 μL PCR reaction contained 12.5 μL Takara CloneAmp HiFi 2X PCR Premix, 1 μL DNA template, 1 μL forward primer, 1 μL reverse primer, and 9.5 μL molecular grade water. Thirty cycles of PCR were performed, with annealing temperatures of 62 °C (WA-UW-074978), 58 °C (WA-UW-082786), or 60 °C (WA-UW-096851), followed by 30 s of extension at 72°C. Five microliters of PCR product was then run on a 1.2% agarose gel in TAE buffer adjacent to the Promega 1 kb Ladder, and visualized with Sigma Aldrich GelRed Nucleic Acid Stain. Amplicons were purified with 0.8× AMPure XP beads and eluted in molecular water, quantified by Qubit (ThermoFisher, Waltham, MA, USA) and Sanger sequenced.

For WA-UW-074978, because the deletion included the ITR, we also performed ddPCR to measure copy number. Primer and probe sets were designed to amplify either a segment of the MPXVgp004/MPXVgp188 gene, which is contained in both ITRs (5′-GACAAAAACAAGCATGCTTCCC-3′; 5′-AGCTTTAGTGACCTGCTCGG-3′; probe: 5′-/56-FAM/TAAACATCCAGTTTTGACACAGT/MGB-NFQ/-3′), or a segment of the F3L gene (5′-CATCTATTATAGCATCAGCATCAGA-3′; 5′-GTAGACCAACGAGGAGGAGTATC-3′; 5′-/56-FAM/TGTAGGCCGTGTATCAGCATCCATT/BHQ1/-3′), which is not part of the ITRs. DNA templates were diluted in water to roughly Ct 28 to enable accurate quantitation. All ddPCR reactions contained 900 nM each of forward and reverse primers, 250 nM probe, 1× ddPCR Supermix for Probes (BioRad, Hercules, CA, USA), and 10µL of template in a 25 µL reaction. ITR ddPCR reactions also contained 0.25 µL of EcoRV restriction enzyme (NEB, Ipswich, MA, USA). Droplet generation was carried out on the BioRad Automated Droplet Generator according to manufacturer instructions, using Automated Droplet Generation Oil for Probes (BioRad). Thermocycling consisted of 95 °C for 10 min, 40 cycles of 94 °C for 30 s and 60 °C for 1 min, 98 °C for 10 min, then a 4 °C hold. Droplets were read on the QX200 (BioRad), and data were analyzed using QuantaSoft Analysis Pro v.1.0.596 (BioRad).

## 3. Results and Discussion

Since July 2022, our laboratory has routinely sequenced MPXV PCR-positive specimens detected via clinical testing, with 207 consensus MPXV genomes determined as of 24 October 2022, contributing to a near real-time understanding of MPXV diversity [24]. However, functional annotation of MPXV genes has lagged, due in large part to the technical challenges associated with culturing MPXV and knocking out genes of interest. Therefore, examination of predicted protein domains and functions in ORFs that can tolerate deletion or disruption by nonsense or frameshift mutations in circulating MPXV can augment our current understanding of MPXV gene functions, essentiality, and adaptation to humans, as well as guide ongoing functional and biochemical studies. Overall, we describe twenty-five clinical specimens containing eleven unique ORF-disrupting mutations. 

**Figure 1:**
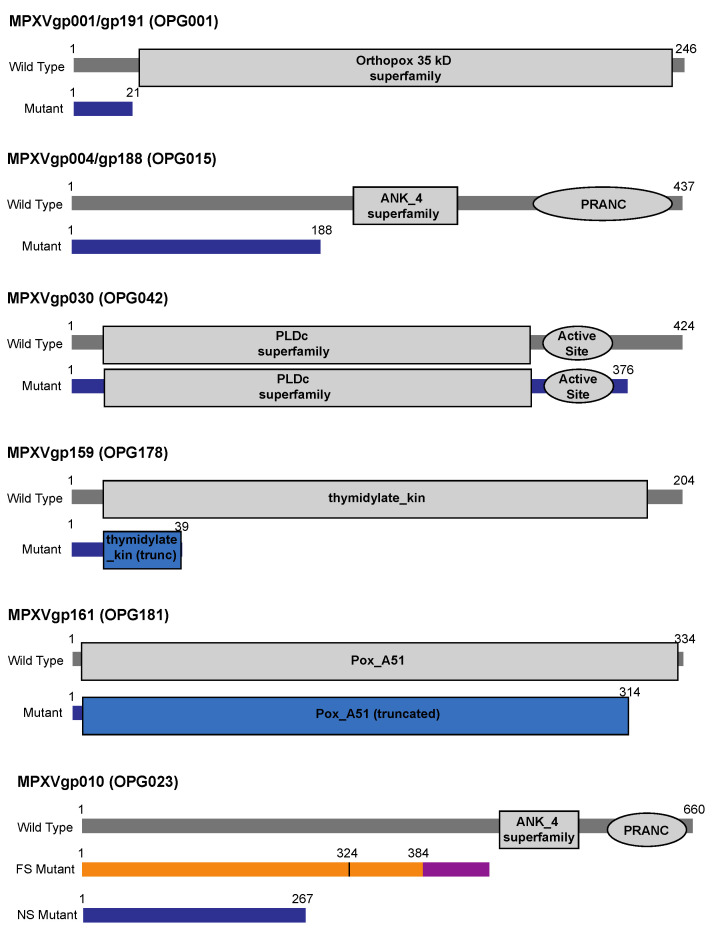
Schematic of predicted architecture of MPXV proteins disrupted by nonsense or frameshift mutations. Wild-type and mutant proteins are shown, with amino acid positions of N and C termini of wildtype, and location of premature stop. Mutants containing nonsense mutations are shown in blue, and domains truncated by the mutation are colored blue. For the mutant of MPXVgp010 frameshifted at residue 384, the alternative C terminus is colored in purple, the appropriate N-terminus is colored in orange, and the M324I mutation is marked.

One nonsense mutation in particular was found repeatedly. In ten samples, including seven samples from five individuals from Washington State (WA-UW-091243, WA-UW-092889, and WA-UW-098497 are lesion swab samples from the same patient) and three samples from Ohio, we saw nonsense mutations in the identical open reading frames MPXVgp004 and MPXVgp188 (both OPG015), which are found in the 5’ and 3’ ITRs, respectively, and encode a protein of unknown function. A conserved domain search on the wild-type protein revealed predicted ankyrin repeat domains spanning residues 221–282 as well as a poxvirus-specific ankyrin repeat motif, a PRANC domain, spanning residues 348–433. All of these predicted ankyrin repeats are excluded from the putative truncated protein product, which in both MPXVgp004 and MPXVgp188 has been truncated after Ile187 due to a C -> T nonsense mutation in the CAG codon encoding Gln188, corresponding to a G -> A transition at nt 5612 and a C -> T transition at nt 191594 relative to the 2022 outbreak reference strain MA001 (ON563414) (Figure 1) [25]. Although the specific function of MPXVgp004/gp188 is not documented, it is one of the eight ankyrin-like proteins in MPXV. MPXV ankyrin-like proteins are known to inhibit host antiviral signaling and inflammatory responses by disrupting activation of NF-κB22, likely via the PRANC domain, which is structurally related to the F-box domains found in the Skp1-Cullin-Fbox (SCF) E3 ubiquitin ligase complex that tags inhibitors of NF-κB activation for degradation. The persistence of a mutated form of a gene whose function is to disrupt one of the primary host antiviral signaling pathways suggests functional redundancy among MPXV ankyrin repeat-containing proteins [26]. 

A nonsense mutation was also detected in three samples in another ORF predicted to encode a protein that attenuates the host immune response. Relative to the MA001 reference, samples WA-UW-085393, and WA-UW-082002 and WA-UW-085241, which were from the same individual, had mutations G1529A and C195677T, which are antisense to each other in the ITR. These paired mutations resulted in nonsense mutation Q22* in the open reading frame MPXVgp001/MPXVgp191 (OPG001) (Figure 1), which encodes the ortholog of vaccinia C23L, an abundantly secreted protein that binds and inhibits host CC and CXC chemokines to modulate the host response [27]. Although the predicted Pfam structure for the conserved Orthopox 35 kDa superfamily gene begins at Ser27, it is plausible that at least partial function of this protein is retained by using an alternative start codon, encoding Met37, although this abrogates the secretion sequence predicted for the full-length protein.

In two lesion swab samples collected from the same patient (WA-UW-088793 and WA-UW-083698), we found a nonsense mutation in MPXVgp159 (OPG178, encoding the 204 aa protein thymidylate kinase, TMPK), after the first 39 amino acids, due to a MA001 C151619T change (Figure 1), certainly resulting in a non-functional protein. Interestingly, although poxvirus TMPK is potentially an attractive drug target [28], its ability to be complemented by the human TMPK is unknown. 

Other nonsense mutations occurred in ORFs encoding proteins of completely unknown function, with little or no supporting literature in any orthopoxvirus. For example, a nonsense mutation was seen in MPXVgp030 (OPG042) in four samples: WA-UW-093570, WA-UW-092113, WA-UW-085088, and WA-UW-084331. A C -> T transition at MA001 nt 25984 results in the premature truncation of MPXVgp030, encoding a predicted phospholipase D-like protein, after Asp376, dropping the last 48 amino acids (Figure 1). Although a Pfam database search revealed that this should not disrupt the predicted active site of the putative enzyme, which is predicted to be formed by residues 320–346, the effects on the structure of the protein are unknown. 

In sample WA-UW-081469, a C -> T transition at MA001 nt 155347 caused nonsense mutation R315* in the open reading frame MPXVgp161 (OPG181), causing the truncation of the final 20 amino acids from the highly conserved C terminus of poxvirus protein A51 (Figure 1), a protein of unknown function. A similar deletion is found in the Vaccinia Ankara strain (NCBI accession AY603355).

**Figure 2:**
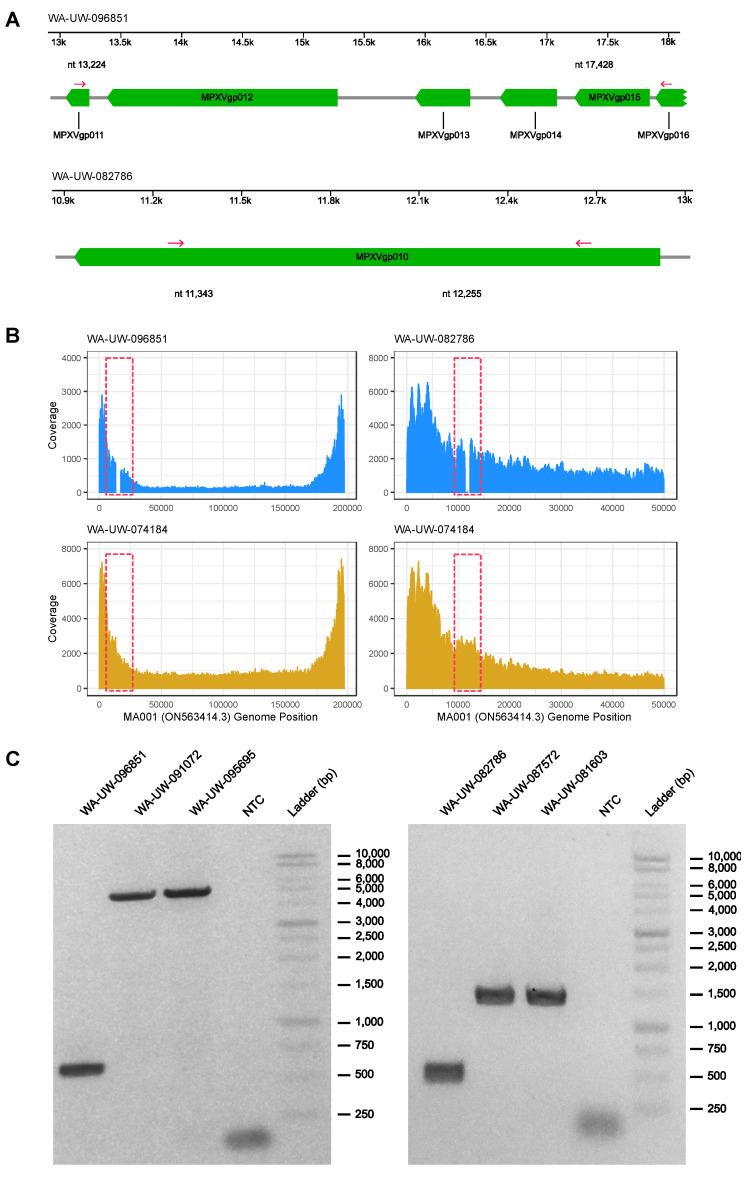
4205 bp deletion in WA-UW-096851 and 913 bp deletion in WA-UW-082786: (**A**) Schematic of 4205 bp deletion in WA-UW-096851 913 bp deletion in WA-UW-082786 (genomic position relative to ON563414.3). Deleted regions are highlighted in blue. Deletion PCR primers (highlighted red) are indicated by arrows; (**B**) Coverage plots with positions shown relative to MA001/ON563414.3 show the drop in coverage in deleted regions in WA-UW-096851 and WA-UW-082786 (highlighted in red dotted box), which are not present in a control sample (WA-UW-074184); (**C**) PCR confirmation of 4205 bp deletion in WA-UW-096851 and 913 bp deletion in WA-UW-082786 compared to other wild-type MPXV samples.

In sample OH-UW-070197, the deletion of MA001 C11764 causes a frameshift after residue Gly384 in MPXVgp010 (OPG023). Like the nonsense mutations introduced in MPXVgp004/MPXVgp188, the mutation eliminates the predicted ankyrin repeat and PRANC/Fbox domains at positions 452–504 and 579–658, respectively, instead encoding a novel 48 amino acid C terminus. Sample OH-UW-070197 also contains a non-synonymous mutation that results in the substitution of Met324 with Ile (Figure 1). In samples WA-UW-082002 and WA-UW-085241, a G12116A (relative to MA001) nonsense mutation upstream of the predicted ankyrin repeat and PRANC/Fbox domains was found. As with nonsense mutation of MPXVgp004/188 (OPG015), it is possible that functional redundancy within ankyrin repeats does not affect the fitness of the mutant strain. Additionally, in sample WA-UW-082786, a 913 bp deletion within MPXVgp010 from nt 11,343 to nt 12,255 (relative to MA001) resulted in a frameshift at Ser222, forming a novel five residue C terminus prior to termination. This mutation would also eliminate the ankyrin repeat and PRANC domains. The mutation was confirmed by PCR (Figure 2). 

In OH-UW-071048, we found an insertion of an additional adenine in the adenine homopolymer at position 128944–128951 of MA001 in the open reading frame MPXVgp135 (OPG151), which encodes the DNA-dependent RNA polymerase subunit rpo132. MPXVgp135 is annotated to start at nt 128941 in MA001. As MPXVgp135 is currently annotated in MA001, an A128944 insertion would result in a frameshift mutation that reduces the gene product to a 6 amino acid residue. Interestingly, the Modified Vaccinia Virus Ankara strain (NCBI Accession AY603355) also has the same insertion and its rpo132 gene is annotated to start 21 nucleotides downstream of A128944 (MA001 nt 128965). In addition, ribosome profiling data of vaccinia virus [29] annotated the translation initiation site at an ATG 24 nt 3’ from the ATG start site annotated in the vaccinia virus refrence genome (NC_006998) and that matches the annotated ATG seen in Modified Vaccinia Virus Ankara noted above. Since rpo132 is an essential gene, we believe the MPXVgp135 open reading frame in OH-UW-071048 most likely starts at MA001 nt 128965 as well. Ribosome profiling data for monkeypox virus in culture will likely help better annotate genes and coding sequences across its genome.

In OH-UW-096851, a 4205 bp deletion from MA001 position nt 13,224 to nt 17,428 was detected by sequencing and confirmed by PCR (Figure 2). This deletion eliminates the start codon of the 64 residue ORF MPXVgp011, as well as MPXVgp012 (OPG025), MPXVgp013 (OPG027), and MPXVgp014 (OPG029). It also creates a novel 11-residue C terminus for MPXVgp015 after Thr141. MPXVgp012, MPXVgp013, MPXVgp014 all encode proteins annotated as type I interferon antagonists; MPXVgp012 has been implicated in the differences in virulence between west and central African MPXV strains [12]. MPXVgp012 also contains a predicted ankyrin repeat domain. MPXVgp015 is predicted to contain a Kelch-like domain involved in ubiquitination, which is truncated by the mutation in OH-UW-096851.

**Figure 3:**
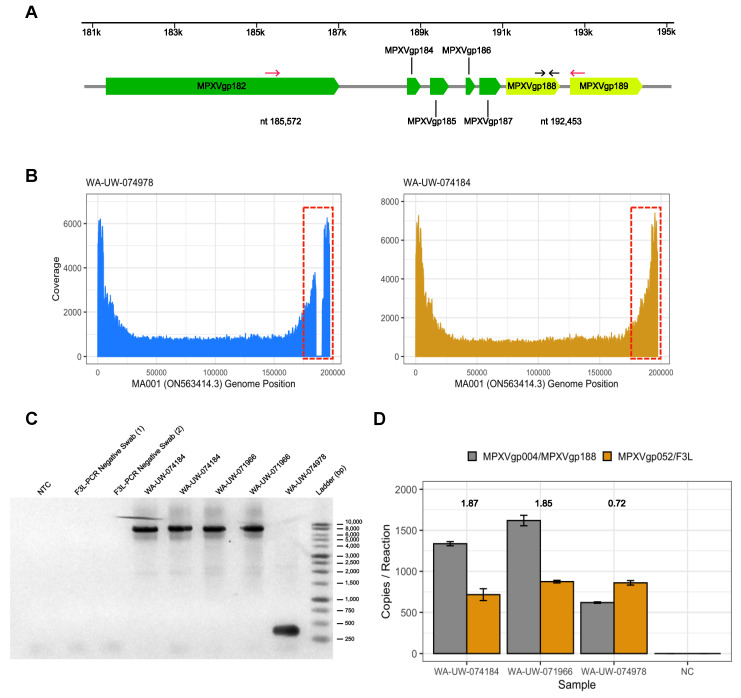
6881 bp deletion in WA-UW-074978 eliminates several ORFs: (**A**) Schematic of deletion of 6881 nucleotides (highlighted blue) from nt 185,572 to 192,453 (relative to ON563414.3) in WA-UW-074978. Genes in the ITR regions are highlighted yellow. Deletion PCR primers (highlighted red) and ddPCR primers (highlighted black) are indicated by arrows; (**B**) Coverage plot with positions shown relative to MA001/ ON563414.3 shows drop in coverage in deleted region in sample WA-UW-074978 (highlighted in red dotted box), which is not present in a control sample (WA-UW-074184) that is known to be intact; (**C**) PCR confirmation of 7 kb deletion in WA-UW-074978 compared to two other wild-type MPXV samples WA-UW-074184 and WA-UW-071966 and two F3L-PCR confirmed MPXV-negative controls; (**D**) Copy numbers of MPXVgp004/MPXVgp188 and F3L in WA-UW-074184, WA-UW-071966, WA-UW-074978, and a F3L-PCR negative control as determined by ddPCR. Ratio of MPXVgp004/MPXVgp188 to F3L locus is indicated above each specimen.

Finally, we found a 6881 bp deletion from MA001 nt 185,572 to nt 192,453 bp in WA-UW-074978 (Figure 3A). Manual inspection of reads revealed a clear drop in coverage of that region of the consensus genome MA001 compared to mapping of reads from another MPXV-positive specimen (Figure 3A,B). The deletion eliminated MPXVgp184, MPXVgp185 (OPG005), MPXVgp186, MPXVgp187 (OPG016), MPXVgp188 (OPG015) and removed the 3′ 1424 bp from MPXVgp182/B21R, resulting in a truncated MPXVgp182/B21R open reading frame (Figure 3A), similar to previous reports [8,30]. The deletion was confirmed by PCR (Figure 3C) and Sanger sequencing. Although the 7kb deletion occurred partially within the 3’ ITR, no genomic rearrangement was detected as has been seen in a similar deletion that did not involve MPXVgp182/B21R [8], as evidenced by a ddPCR copy number ratio of MPXVgp004/188 to F3L in the mutant of 0.72, in contrast to ratios of 1.87 and 1.85 seen in MPXV strains without the deletion (Figure 3D). This pattern is consistent with the loss of only the 3’ ITR MPXVgp188 (identical to 5’ ITR MPXVgp004) in WA-UW-074978. WA-UW-074978 was sequenced from a lesion swab with a MPXV qPCR Ct of 22.2, which matches the strong viral loads typically seen in MPXV-positive specimens from lesion swabs [14]. Intriguingly, this individual also had MPXV-PCR-positive specimen taken from a concomitant knee effusion that repeatedly tested positive at low viral loads (Ct 38.5 and Ct 34.5 after 10-fold concentration).

The putative functions of some of the deleted genes in WA-UW-074978 were reviewed by Shchelkunov et al. in an overview of poxvirus orthologs [31]. MPXVgp184 is expected to be an inhibitor of apoptosis [31,32], consistent with its Pfam-predicted Bax-1 Inhibitor-like domain. MPXVgp187 is a secreted MHC-I-like protein that binds to NKG2D on NK cells and prevents them from binding their ligands on infected cells [33]. As discussed above, MPXVgp004/188 contains ankyrin-like repeats [31]. The function of MPXVgp185 is unknown and a search of the Pfam database reveals only that it is a conserved poxvirus protein, while MPXVgp186, a predicted 74 aa ORF, has no predicted conserved domains. MPXVgp182/B21R is an immunogenic surface glycoprotein that was previously proposed as an essential antibody target and regulator of T-cell function [34]. Notably, the deleted C terminal region of MPXVgp182/B21R in WA-UW-074978 harbors epitopes to which the vast majority of MPX patients were reactive in a serum screen to develop novel MPXV-specific serodiagnostics [13], raising the possibility that deletion of this region represents immunoevasion by MPXV. MPXVgp182/B21R-deleted variants of MPXV demonstrated milder disease and lower viremia in non-human primate models compared to wild type MPXV [34]. However, this specimen had a relatively strong viral load with a Ct 22.2.

A 15 kb deletion spanning from MPXVgp182/B21R to the 3′ ITR region has been reported in a sample collected in Florida State, USA. Compared to the 7 kb deletion in WA-UW-074978, the 15 kb deletion truncates most of MPXVgp182/B21R and eliminates the other three open reading frames in the 3’ ITR region—MPXVgp189 (OPG003), MPXVgp190 (OPG002), and MPXVgp191 (OPG001) [10]. Another strain that includes deletion of MPXVgp184 to MPXVgp187 has previously been reported in Germany, however, this deletion was significantly smaller at 2048 nt and involved an 856 nt duplication from the 5’ region, but did not disrupt the MPXVgp182/B21R open reading frame [8]. The 856 nt duplication includes the full length of the MPXVgp005 open reading frame as well as 502 nt before and 135 nt after. 

**Figure 4:**
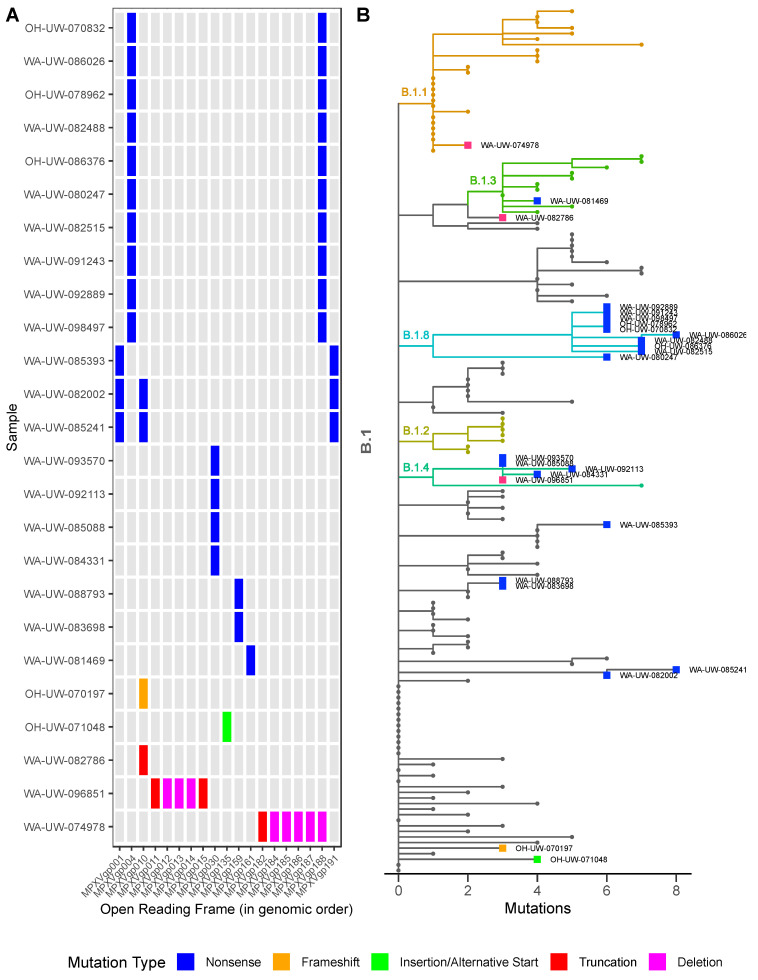
Characterization and phylogenetic analysis of MPXV variants: (**A**) Map of mutation types and affected genes in MPXV variants by mutation type. (**B**) Phylogenetic analysis of MPXV variants along with 129 other samples sequenced by the University of Washington Virology lab by mutation type. All variants belong to the 2022 outbreak lineage B.1.

Finally, we examined all detected ORF-disrupting mutations (Figure 4A) in the context of the MPXV phylogeny (Figure 4B). Phylogenetic analysis revealed that all MPXV variants belong to the 2022 monkeypox outbreak B.1 lineage. Outside of the 7 kb deletion, WA-UW-074978 is only two mutations diverged from the B.1 lineage, while all other variants with nonsense and frameshift mutations are three to eight mutations different from the B.1 lineage, suggesting that all variants evolved from the current outbreak and do not represent the introduction of divergent strains. All strains harboring the MPXVgp004/188 nonsense mutation belong to B.1.8, therefore it is highly likely the mutations are fixed and potentially transmissible. Mutation allele frequencies from multiple sequencing efforts are documented in Supplementary Table S2. All the nonsense mutations are G -> A and C -> T. This biased mutation pattern could be an indication of APOBEC3 editing activities as has been previously reported [6,7].

With the exception of the viral strain containing insertion and premature stop in rpo132, which we believe may instead make use of an alternative start codon at Met9 as currently annotated, there is no evidence that any of the disrupted genes presented in this study are in essential genes. Rather, our results are consistent with previous reports of most mutations occurring in the highly labile ITRs and adjacent regions that encode genes involved in the exquisite fine-tuning of host tropism characteristic of poxviruses. Among the genes affected by mutation in this study, two (MPXVgp004/188 and MPXVgp010) encode ankyrin repeat-containing proteins that could interact with the NF-κB pathway, one encodes a chemokine antagonist (MPXVgp001/191), one encodes an NKG2D antagonist (MPXVgp187), one encodes an apoptosis inhibitor (MPXVgp184), and one is in a thymidylate kinase (MPXVgp161) of unknown essentiality. The remainder of ORFs affected are of unknown function, except for MPXVgp182, which is a surface glycoprotein of unknown function, but is highly immunogenic. Therefore, the evidence in our limited dataset suggests ORF disruption in MPXV is limited to non-essential genes, particularly those known or predicted to directly interact with the host immune system. 

Additionally, our findings have important implications for diagnostics as MPXVgp182/B21R has been targeted as a potential monkeypox virus serological antigen since it is highly immunogenic and has been lost in contemporary vaccinia strains used for vaccination, therefore seroreactivity to MPXVgp182/B21R is specific to prior infection with MPXV [13]. Furthermore, the repeated modifications found in peri-ITR regions in poxviruses indicate that these regions are not the best diagnostic targets for PCR or serology testing.

## Data Availability

Sequencing reads and consensus assemblies are available from NCBI BioProject PRJNA862948.

## Supplementary Materials

The following supporting information can be downloaded at: https://www.mdpi.com/article/10.3390/v14112393/s1, Table S1: Sequence Read Archive (SRA) and GenBank accessions for MPXV sequencing raw reads and consensus genomes with indicated mutations; Table S2. Monkeypox virus variants mutation allele frequencies calculated using pysamstats v1.1.2.
