# Supplementary material for: ORF-Interrupting Mutations in Monkeypox Virus Genomes from Washington and Ohio, 2022
Source: Viruses. 2022 Oct 29;14(11):2393. doi: 10.3390/v14112393 (PMC9695478; doi:10.3390/v14112393)
Supplement: Supplementary file 1 [file viruses-14-02393-s001.zip › SuppTableS1_MPXVdeletion_revision_updated102522.pdf]

Supplementary Materials

Supplementary Table S1. Sequence Read Archive (SRA) and GenBank accessions for MPXV sequencing raw reads and consensus genomes with indicated mutations. Mutation types and genomic positions relative to ON563414.3 are indicated. SRA accessions for raw reads from the resequencing of variants are indicated by asterisks.

Sample	GenBank Accession	SRA Accession	Mutation Type
WA-UW-074978	OP434519.1	SRR20913436<br>SRR21620332*	Deletion 185572-192453
WA-UW-096851	OP681776.1	SRR21966445<br>SRR22019271*	Deletion 13224-17428
WA-UW-082786	OP650027.1	SRR21897921<br>SRR22019272*	Deletion 11343-12255
OH-UW-070197	OP442936.1	SRR21236090<br>SRR21620337*	Deletion C11764
OH-UW-070832	OP442937.1	SRR20913441<br>SRR21620331*	G5612A, C191594T
OH-UW-071048	OP442938.1	SRR21598457<br>SRR21620329*	Insertion A128943
OH-UW-078962	OP442939.1	SRR21236093<br>SRR21620338*	G5612A, C191594T
OH-UW-086376	OP442940.1	SRR21236113<br>SRR21620340*	G5612A, C191594T
WA-UW-082488	OP442941.1	SRR21236117<br>SRR21620341*	G5612A, C191594T
WA-UW-084331	OP442942.1	SRR21236107<br>SRR21620330*	C25984T
WA-UW-086026	OP442943.1	SRR21236097<br>SRR21620339*	G5612A, C191594T
WA-UW-081469	OP442944.1	SRR21524980<br>SRR21620335*	C155347T
WA-UW-083698	OP442945.1	SRR21524973<br>SRR21620333*	C151619T
WA-UW-085393	OP442946.1	SRR21524975<br>SRR21620336*	G1529A, C195677T
WA-UW-088793	OP442947.1	SRR21524979<br>SRR21620334*	C151619T
WA-UW-080247	OP628437.1	SRR21887143<br>SRR22019269*	G5612A, C191594T
WA-UW-085241	OP628463.1	SRR21887132<br>SRR22019268*	G1529A, G12116A,<br>C195677T
WA-UW-093570	OP628490.1	SRR21887131<br>SRR22019267*	C25984T
WA-UW-082515	OP628447.1	SRR21887120<br>SRR22019266*	G5612A, C191594T

WA-UW-082002	OP628445.1	SRR21887118<br>SRR22019265*	G1529A, G12116A,<br>C195677T
WA-UW-091243	OP681773.1	SRR21966448<br>SRR22019264*	G5612A, C191594T
WA-UW-092889	OP681774.1	SRR21966447<br>SRR22019263*	G5612A, C191594T
WA-UW-098497	OP681775.1	SRR21966446<br>SRR22019262*	G5612A, C191594T
WA-UW-092113	OP681777.1	SRR21966444<br>SRR22019270*	C25984T
WA-UW-085088	OP681772.1	SRR21966449<br>SRR22019273*	C25984T
WA-UW-074184	OP169341.1	SRR20913437	
WA-UW-071966	OP169344.1	SRR20913433	
WA-UW-091072	OP715788.1	SRR22033832	
WA-UW-095695	OP715789.1	SRR22033831	
WA-UW-087572	OP628476.1	SRR21887146	
WA-UW-081603	OP628444.1	SRR21887145	
WA-UW-073909	OP055800.1	SRR20653196	
WA-UW-078327	OP055801.1	SRR20653195	
Unknown-UW-077794	OP055802.1	SRR20653194	
Unknown-UW-073599	OP055803.1	SRR20653193	
WA-UW-076724	OP055804.1	SRR20653192	
Unknown-UW-076991	OP055805.1	SRR20653191	
WA-UW-079141	OP055806.1	SRR20653190	
WA-UW-074372	OP055807.1	SRR20653189	
WA-UW-075986	OP055808.1	SRR20653188	
WA-UW-076082	OP055809.1	SRR20653187	
WA-UW-073974	OP123040.1	SRR20731575	
WA-UW-070134	OP123041.1	SRR20731574	
WA-UW-077622	OP123042.1	SRR20731573	
WA-UW-077836	OP123043.1	SRR20731572	
WA-UW-071246	OP123044.1	SRR20736996	
WA-UW-076225	OP123045.1	SRR20736995	
WA-UW-078870	OP123046.1	SRR20736992	
WA-UW-071121	OP123047.1	SRR20736991	
WA-UW-076861	OP123048.1	SRR20736990	
WA-UW-073669	OP123049.1	SRR20736989	
WA-UW-076854	OP123050.1	SRR20736988	
OH-UW-070426	OP123051.1	SRR20736987	
OH-UW-078654	OP123052.1	SRR20736986	
OH-UW-071686	OP123053.1	SRR20736985	

OH-UW-077163	OP123054.1	SRR20736994	
OH-UW-070822	OP123055.1	SRR20736993	
WA-UW-070499	OP169336.1	SRR20913445	
OH-UW-079847	OP169337.1	SRR20913444	
OH-UW-074058	OP169338.1	SRR20913440	
OH-UW-076485	OP169339.1	SRR20913439	
WA-UW-074224	OP169340.1	SRR20913438	
OH-UW-072752	OP169342.1	SRR20913435	
OH-UW-075598	OP169343.1	SRR20913434	
WA-UW-075687	OP169345.1	SRR20913443	
WA-UW-072839	OP169346.1	SRR20913442	
WA-UW-079401	OP184760.1	SRR20973040	
WA-UW-078603	OP184761.1	SRR20973039	
WA-UW-074949	OP184762.1	SRR20973038	
WA-UW-076773	OP184763.1	SRR20973037	
WA-UW-074932	OP184764.1	SRR20973036	
WA-UW-074988	OP184765.1	SRR20973035	
WA-UW-089015	OP257243.1	SRR21236108	
WA-UW-080268	OP257244.1	SRR21236106	
WA-UW-088225	OP310068.1	SRR21236105	
WA-UW-084909	OP257245.1	SRR21236104	
WA-UW-083578	OP257246.1	SRR21236103	
WA-UW-088432	OP257247.1	SRR21236102	
WA-UW-087424	OP257248.1	SRR21236101	
WA-UW-081786	OP257249.1	SRR21236100	
WA-UW-087527	OP257250.1	SRR21236099	
WA-UW-081272	OP257251.1	SRR21236096	
WA-UW-085937	OP257252.1	SRR21236095	
WA-UW-088081	OP257253.1	SRR21236094	
OH-UW-073812	OP257254.1	SRR21236092	
OH-UW-073319	OP257255.1	SRR21236091	
OH-UW-082260	OP257256.1	SRR21236089	
WA-UW-080706	OP257257.1	SRR21236088	
WA-UW-086040	OP257258.1	SRR21236086	
WA-UW-085684	OP257259.1	SRR21236085	
WA-UW-087006	OP257260.1	SRR21236084	
WA-UW-084325	OP257261.1	SRR21236083	
WA-UW-087619	OP257262.1	SRR21236082	
WA-UW-089987	OP257263.1	SRR21236081	

WA-UW-080976	OP257264.1	SRR21236080	
WA-UW-088152	OP310069.1	SRR21236079	
WA-UW-089183	OP257265.1	SRR21236078	
WA-UW-083179	OP257266.1	SRR21236077	
WA-UW-084449	OP257267.1	SRR21236142	
OH-UW-086011	OP310038.1	SRR21236120	
WA-UW-080617	OP310039.1	SRR21236109	
WA-UW-088656	OP310040.1	SRR21236098	
WA-UW-080185	OP310041.1	SRR21236087	
WA-UW-089266	OP310042.1	SRR21236076	
WA-UW-086841	OP310043.1	SRR21236075	
WA-UW-083888	OP310044.1	SRR21236074	
WA-UW-080043	OP310045.1	SRR21236141	
WA-UW-083914	OP310046.1	SRR21236140	
WA-UW-087301	OP310047.1	SRR21236139	
WA-UW-088871	OP310048.1	SRR21236138	
OH-UW-085556	OP310049.1	SRR21236137	
OH-UW-085701	OP310050.1	SRR21236136	
WA-UW-082029	OP310051.1	SRR21236135	
WA-UW-081935	OP310052.1	SRR21236134	
WA-UW-082670	OP310053.1	SRR21236133	
WA-UW-088213	OP310054.1	SRR21236132	
WA-UW-086360	OP310055.1	SRR21236130	
WA-UW-089437	OP310056.1	SRR21236129	
WA-UW-089714	OP310057.1	SRR21236128	
WA-UW-087349	OP310058.1	SRR21236127	
OH-UW-089521	OP310059.1	SRR21236126	
OH-UW-082225	OP310060.1	SRR21236125	
WA-UW-088764	OP310061.1	SRR21236124	
WA-UW-089836	OP310062.1	SRR21236123	
WA-UW-082810	OP310063.1	SRR21236122	
WA-UW-082497	OP310064.1	SRR21236121	
WA-UW-088973	OP310065.1	SRR21236119	
WA-UW-089152	OP310066.1	SRR21236118	
WA-UW-082625	OP310067.1	SRR21236116	
WA-UW-083953	OP328307.1	SRR21236131	
WA-UW-083083	OP328308.1	SRR21236115	
WA-UW-089142	OP328309.1	SRR21236114	
WA-UW-088725	OP328310.1	SRR21236112	

WA-UW-088258	OP328311.1	SRR21236111	
WA-UW-087564	OP328312.1	SRR21236110	
WA-UW-080292	OP392536.1	SRR21524966	
WA-UW-080834	OP392537.1	SRR21524964	
WA-UW-083584	OP392543.1	SRR21524963	
OH-UW-089384	OP392534.1	SRR21524962	
WA-UW-085171	OP392546.1	SRR21524961	
WA-UW-087094	OP392548.1	SRR21524960	
WA-UW-080100	OP392535.1	SRR21524985	
OH-UW-085672	OP392533.1	SRR21524984	
OH-UW-080114	OP392532.1	SRR21524983	
WA-UW-088960	OP392553.1	SRR21524982	
WA-UW-083506	OP392542.1	SRR21524981	
WA-UW-081714	OP392538.1	SRR21524978	
WA-UW-082880	OP392541.1	SRR21524977	
WA-UW-087104	OP392549.1	SRR21524976	
WA-UW-083781	OP392544.1	SRR21524974	
WA-UW-085462	OP392547.1	SRR21524972	
WA-UW-088325	OP392552.1	SRR21524971	
OH-UW-071356	OP392531.1	SRR21524970	
WA-UW-088092	OP392551.1	SRR21524968	
WA-UW-082770	OP392540.1	SRR21524969	
WA-UW-087336	OP392550.1	SRR21524987	
WA-UW-082215	OP392539.1	SRR21524967	
WA-UW-084148	OP392545.1	SRR21524986	
