# Supplementary material for: ORF-Interrupting Mutations in Monkeypox Virus Genomes from Washington and Ohio, 2022
Source: Viruses. 2022 Oct 29;14(11):2393. doi: 10.3390/v14112393 (PMC9695478; doi:10.3390/v14112393)
Supplement: Supplementary file 1 [file viruses-14-02393-s001.zip › SuppTableS2_landscape_MPXVdeletion_revision_updated102422.pdf]

Supplementary Materials

Supplementary Table S2. Monkeypox virus variants mutation allele frequencies calculated using pysamstats v1.1.2 (https://github.com/alimanfoo/pysamstats/releases/tag/v1.1.2) with a minimum base quality phred score of 20. ON563414.3 was used as reference. Resequenced samples are indicated by asterisks.

sample	pos	ref	base counts	matches	mismatches	deletions	insertions	A	C	T	G	mismatch/ indel frequency
OH-UW-070197	11764	C	2734	162	5	2567	0	0	162	5	0	0.9389
OH-UW-070197*	11764	C	3126	195	7	2924	0	0	195	7	0	0.9354
OH-UW-070832	5612	G	3475	0	3475	0	0	3475	0	0	0	1.0000
OH-UW-070832*	5612	G	540	1	539	0	0	539	0	0	1	0.9981
OH-UW-070832	191594	C	3471	2	3469	0	0	0	2	3469	0	0.9994
OH-UW-070832*	191594	C	515	0	515	0	0	0	0	515	0	1.0000
OH-UW-071048	128943	G	121	117	4	0	114	4	0	0	117	0.9421
OH-UW-071048*	128943	G	299	296	3	0	258	3	0	0	296	0.8629
OH-UW-078962	5612	G	441	0	441	0	0	441	0	0	0	1.0000
OH-UW-078962*	5612	G	586	0	586	0	0	586	0	0	0	1.0000
OH-UW-078962	191594	C	452	0	452	0	0	0	0	452	0	1.0000
OH-UW-078962*	191594	C	605	1	604	0	0	0	1	604	0	0.9983
OH-UW-086376	5612	G	1874	0	1874	0	0	1874	0	0	0	1.0000
OH-UW-086376*	5612	G	1856	0	1856	0	0	1856	0	0	0	1.0000
OH-UW-086376	191594	C	1867	0	1867	0	0	0	0	1866	1	1.0000
OH-UW-086376*	191594	C	1954	0	1954	0	0	0	0	1954	0	1.0000
WA-UW-081469	155347	C	18	2	16	0	0	0	2	16	0	0.8889
WA-UW-081469*	155347	C	14	5	9	0	0	0	5	9	0	0.6429

Supplementary Table S2 Cont.

sample	position	ref	base counts	matches	mismatches	deletions	insertions	A	C	T	G	mismatch/ indel frequency
WA-UW-082488	5612	G	880	1	879	0	0	879	0	0	1	0.9989
WA-UW-082488*	5612	G	864	0	864	0	0	864	0	0	0	1.0000
WA-UW-082488	191594	C	868	0	868	0	0	0	0	868	0	1.0000
WA-UW-082488*	191594	C	838	0	838	0	0	0	0	838	0	1.0000
WA-UW-083698	151619	C	137	0	137	0	0	0	0	137	0	1.0000
WA-UW-083698*	151619	C	117	0	117	0	0	0	0	117	0	1.0000
WA-UW-084331	25984	C	101	0	101	0	0	0	0	101	0	1.0000
WA-UW-084331*	25984	C	213	2	211	0	0	0	2	211	0	0.9906
WA-UW-085393	1529	G	496	1	495	0	0	495	0	0	1	0.9980
WA-UW-085393*	1529	G	228	0	228	0	0	228	0	0	0	1.0000
WA-UW-085393	195677	C	484	1	483	0	0	0	1	483	0	0.9979
WA-UW-085393*	195677	C	232	1	231	0	0	0	1	231	0	0.9957
WA-UW-086026	5612	G	636	257	379	0	0	379	0	0	257	0.5959
WA-UW-086026*	5612	G	993	371	622	0	0	622	0	0	371	0.6264
WA-UW-086026	191594	C	620	242	378	0	0	0	242	378	0	0.6097
WA-UW-086026*	191594	C	1020	382	638	0	0	0	382	638	0	0.6255
WA-UW-088793	151619	C	82	0	82	0	0	0	0	82	0	1.0000
WA-UW-088793*	151619	C	72	0	72	0	0	0	0	72	0	1.0000

Supplementary Table S2 Cont.

sample	position	ref	base counts	matches	mismatches	deletions	insertions	A	C	T	G	mismatch/ indel frequency
WA-UW-080247	191594	C	2071	0	2071	0	0	0	0	2070	1	1.0000
WA-UW-080247*	191594	C	475	0	475	0	0	0	0	475	0	1.0000
WA-UW-080247	5612	G	2097	0	2097	0	0	2097	0	0	0	1.0000
WA-UW-080247*	5612	G	465	2	463	0	0	463	0	0	2	0.9957
WA-UW-082002	12116	G	1813	0	1813	0	0	1813	0	0	0	1.0000
WA-UW-082002*	12116	G	901	0	901	0	0	901	0	0	0	1.0000
WA-UW-082002	1529	G	3954	2	3952	0	0	3952	0	0	2	0.9995
WA-UW-082002*	1529	G	2362	1	2361	0	0	2361	0	0	1	0.9996
WA-UW-082002	195677	C	3970	0	3970	0	0	0	0	3970	0	1.0000
WA-UW-082002*	195677	C	2395	1	2394	0	0	0	1	2394	0	0.9996
WA-UW-082515	191594	C	731	0	731	0	0	0	0	731	0	1.0000
WA-UW-082515*	191594	C	297	0	297	0	0	0	0	297	0	1.0000
WA-UW-082515	5612	G	746	0	746	0	0	746	0	0	0	1.0000
WA-UW-082515*	5612	G	311	1	310	0	0	310	0	0	1	0.9968
WA-UW-085088	25984	C	20	0	20	0	0	0	0	20	0	1.0000
WA-UW-085088*	25984	C	15	0	15	0	0	0	0	15	0	1.0000
WA-UW-085241	12116	G	227	0	227	0	0	227	0	0	0	1.0000
WA-UW-085241*	12116	G	63	0	63	0	0	63	0	0	0	1.0000

Supplementary Table S2 Cont.

sample	position	ref	base counts	matches	mismatches	deletions	insertions	A	C	T	G	mismatch/ indel frequency
WA-UW-085241	1529	G	856	0	856	0	0	856	0	0	0	1.0000
WA-UW-085241*	1529	G	318	1	317	0	0	317	0	0	1	0.9969
WA-UW-085241	195677	C	825	0	825	0	0	0	0	825	0	1.0000
WA-UW-085241*	195677	C	346	0	346	0	0	0	0	346	0	1.0000
WA-UW-091243	191594	C	1355	1	1354	0	0	0	1	1354	0	0.9993
WA-UW-091243*	191594	C	1115	0	1115	0	0	0	0	1115	0	1.0000
WA-UW-091243	5612	G	1352	0	1351	1	0	1351	0	0	0	0.9993
WA-UW-091243*	5612	G	1092	0	1092	0	0	1092	0	0	0	1.0000
WA-UW-092113	25984	C	250	0	250	0	0	0	0	250	0	1.0000
WA-UW-092113*	25984	C	27	0	27	0	0	0	0	27	0	1.0000
WA-UW-092889	191594	C	760	0	760	0	0	0	0	760	0	1.0000
WA-UW-092889*	191594	C	779	0	779	0	0	0	0	779	0	1.0000
WA-UW-092889	5612	G	751	0	751	0	0	751	0	0	0	1.0000
WA-UW-092889*	5612	G	783	0	783	0	0	783	0	0	0	1.0000
WA-UW-093570	25984	C	23	0	23	0	0	0	0	23	0	1.0000
WA-UW-093570*	25984	C	23	0	23	0	0	0	0	23	0	1.0000
WA-UW-098497	191594	C	98	0	98	0	0	0	0	98	0	1.0000
WA-UW-098497*	191594	C	99	0	99	0	0	0	0	99	0	1.0000
WA-UW-098497	5612	G	94	0	94	0	0	94	0	0	0	1.0000
WA-UW-098497*	5612	G	106	0	106	0	0	106	0	0	0	1.0000
